# Winter Residence for Consumptive Patients

**Published:** 1833

**Authors:** 


### Art. XVII. Winter Residence for Consumptive Patients.

We beg leave to direct the attention of the reader, to the remarks on this subject, contained in p. p. 336–8 of this number. From previous information we were prepared to agree in the recommendation of Florida, as a winter retreat for the infirm, in preference to any other part of this continent, or even the island of Cuba. New Orleans, to which many in the West direct their attention, is too cool and humid; while Cuba, although unexceptionable in climate, is highly objectionable in its political and social character.

